# Impact of social separation during pregnancy on the manifestation of defensive behaviors related to generalized anxiety and panic throughout offspring development

**DOI:** 10.1371/journal.pone.0185572

**Published:** 2017-10-16

**Authors:** Flaviane Cristina de Brito Guzzo Soliani, Rafael Cabbia, Matheus Fitipaldi Batistela, Amarylis Garcia Almeida, Vinícius Dias Kümpel, Luiz Yamauchi Junior, Telma Gonçalves Carneiro Spera de Andrade

**Affiliations:** 1 Laboratory of Physiology, UNESP–Univ Estadual Paulista, Assis, São Paulo, Brazil; 2 Department of Biological Science, Laboratory of Physiology, UNESP–Univ Estadual Paulista, Assis, São Paulo, Brazil; Radboud University Medical Centre, NETHERLANDS

## Abstract

The multiple insecurities, anatomical, physiological and psychological changes arising from the gestational period can generate an overload of stress in the mother and cause disturbances in the offspring, affecting it throughout its development. The existing analysis linking prenatal stress and offspring’s anxiety have divergent results, being limited as to gestational week, type of stressor and age of progeny’s assessment. Social separation has been described as a stressor that causes increase in anxiety. Thus, the present study evaluated the effects of social separation applied in one of the three gestational weeks of rat dams on the manifestation of the defensive behaviors related to generalized anxiety disorder and panic in the Elevated T Maze of the male progeny in three stages of development (1, 3 or 6 months of life). It was found, in the offspring of grouped (control) dams, increased behaviors associated with generalized anxiety disorder and a reduction of panic-like behaviors throughout development. For animals whose dams were socially separated during pregnancy, the most critical period of exposure was the 2^nd^ gestational week, which affected the acquisition of aversive memory, demonstrated by the impairment on learning of avoidances of the offspring in all ages evaluated. Stressor exposure in this week also increased the avoidances, related to generalized anxiety of progeny in the 1^st^ month and decreased escapes, related to panic in the 3^rd^ month of life and, at the age of 6 months old, an inverse situation, with the reduction of the defensive behaviors associated to generalized anxiety disorder. The results show that, when assessing effects of prenatal stress on the manifestation of anxiety, not only the period of exposure is important, but also the age of offspring assessed.

## Introduction

It has become increasingly clear that the interaction between genes and environment determines the functional development of an organism. Due to its rapid development, the fetus is particularly vulnerable to disturbances in its hormonal milieu [1]. Emotional or physical environmental adversity experienced by the mother during pregnancy can influence the growth of the fetus, affecting its physical and mental well-being throughout life [2].

However, the manifestation of psychological disorders cannot always be explained by genetic factors, environmental or postnatal life story. It is known that the stress suffered by the embryo or fetus during gestation, i.e., prenatal stress, can cause pathologies [1]. Although the mechanisms by which prenatal stress affects the progeny have not been fully established yet, studies indicate its relationship with the action of catecholamines released due to autonomic activation, decreasing oxygenation and the supply of basic nutrients to the fetus [3, 4], as well as the exposure of the embryo or fetus to glucocorticoids [1, 5], which culminates in the modification of hypothalamic-pituitary-adrenal (HPA) axis’ reactivity in the offspring [6–9]. Evidences also indicate that prenatal stress can alter other neuroendocrine circuitry, such as serotonergic [10–13], noradrenergic [7], GABAergic [14, 15], glutamatergic [15, 16], and of oxytocin and vasopressin [17], indicating direct changes of these systems or indirectly, modulated by the alterations in HPA axis of progeny, via prenatal stress.

Among the various effects observed due to prenatal stress, notably are behavioral changes, such as increase of emotionality [18, 19], depressive behavior [18], increased incidence of attention deficit hyperactivity disorder [20], corticolimbic system deregulation and increased response to fear [21], increased incidence of schizophrenia [22, 23] and increased anxiety-like behaviors [2, 11, 12, 15, 24, 25]. The latter effect is rather controversial, since while some studies have found a positive relationship between prenatal stress and increased anxiety in male offspring, others did not observe the same result [26–29].

In 2008, Marta Weinstock [30] demonstrated in a literature review that the effects caused by prenatal stress depend on the gestational stage in which it is applied, type, intensity and duration of stress, as well as of the species used (mice or rats). Even their strain must be take in account, since differences on the manifestation of anxiety of different strains of rats have been described [31]. In addition, there are rare investigations that have focused on the analysis of anxiety’s manifestation at different stages of offspring’s life. A fact to be highlighted is that most stressors used (e.g., foot or tail shocks, saline injection, restriction for prolonged periods, exposure to bright light, among others) do not simulate the reality experienced by mothers in the human species and not even among the rodents themselves.

Farther, most studies that evaluated the effects of prenatal stress on the manifestation of anxiety in the offspring used the Elevated Plus-Maze (EPM) [32], one of most employed model to study anxiety in rodents. Only one study [24] evaluated prenatal stress effects using the Elevated T-Maze (ETM), a test developed by Graeff and colleagues [33, 34], derived from EPM and also based on the innate fear of rats to high and open locations [35]. The advantage of ETM is the discrimination of two types of anxiety: generalized anxiety disorder and panic [33, 34, 36–39].

It is known that rats and mice are sociable animals, i.e., they live in groups in nature. In most laboratories, they are also kept in this condition, usually in colonies consisting from 4 to 6 animals per box or cage. In fact, social separation has been described as a potent chronic stressor that causes increased anxiety in rats [40, 41], modifying serotonergic neurotransmission in pathways related to this disorder [40], as well as changes on HPA axis activity. In the latter regard, results found for rodents are contradictory: some researchers evidenced increased and others decreased or no alterations in the activity of HPA axis, as shown in the review of Hawkley and colleagues [42]. However, these differences can be attributed to the different species used, the nature of social isolation applied, age of animals and length of isolation [42]. It is worth to mention that both terms ‘social separation’ and ‘social isolation’ have been used in scientific researches. But we understand that social separation would be a deprivation of physical contact only, keeping auditory and olfactory contact, i.e., animals would remain alone in their boxes or cages, but inside the same room. In social isolation animals would be allocated alone in their boxes and also in different rooms. Nevertheless, what is seen is a misunderstanding between these two terms, since many researchers use the word ‘isolation’ but keep animals in the same room, allowing auditory and olfactory contact among them.

Even though social separation is characterized as a stressor, curiously, in the vast majority of studies of prenatal stress, the female was deprived of social contact throughout gestation, i.e., remained alone in its box or cage [2, 5, 9–12, 22, 24, 25, 29, 43].

Thus, the present study’s aim was to evaluate, in ETM, the manifestation of the defensive behaviors related to generalized anxiety disorder and panic in the offspring whose dams were socially separated (or not) in different moments of pregnancy, identifying which stage of the gestational period was more critical for progeny’s postnatal manifestation of these disorders, and also in which phase of postnatal development they were manifested. A more comprehensive approach is that the experiences of the dam would cause neuroendocrine changes that would remain quiescent until a moment of offspring’s life in which there was demand for the systems involved.

## Materials and methods

The study was approved by local Ethics Committee on the Use of Animals of the UNESP–Univ Estadual Paulista (FCL- Assis) (CEUA 011/2012). All procedures were conducted in accordance with international ethical standards concerning animal experimentation.

### Dams

Virgin female *Wistar* rats with an average age of 75 days old, from UNESP Central Vivarium (Botucatu/SP), were grouped (5 rats) in polypropylene boxes (32 x 38 x 18 cm) using sawdust as bedding material in Female’s Vivarium of Physiology Laboratory, maintained under controlled conditions of temperature (21°C ± 2°C), lighting (50 lux at the center of the room and 12:12 hours light-dark cycle, with lights on at 07:00 am), receiving chow and water *ad libitum*. The animals were handled only during box exchange and in specific moments of the experiment.

### Mating

After a minimum period of 7 days of setting in the Vivarium of Physiology Laboratory, the estrous cycle of rats was monitored by vaginal smear. When presented proestrus or estrus, female rats were individually allocated with an experienced male (one couple per box). The criterion for statement of mating and determination of gestational day (GD) 1 was the presence of vaginal plug. When not found, vaginal smear was taken to observe the presence of sperm. After mating, all pregnant female rats (dams) were relocated in their boxes, remaining grouped (5 per box) or not, according to each experimental group.

### Social separation of dams

Dams were socially separated in one of the 3 gestational weeks: 1^st^ week (GD 1 to 7), 2^nd^ week (GD 8 to 14) or 3^rd^ week (GD 15 to 21). In this context, dams, which were grouped into 5 animals per box, were separated into 1 animal per box during the designated gestational week, with all the boxes kept in the same vivarium. Control groups (a total of 3: one for each gestational week) were constituted by dams grouped in 5 animals per box. At the end of the designated gestational week, dams were assessed in the EPM [32], in order to verify if social separation caused alterations in their anxiety’s profile (see the results in [44]). After assessment in the EPM, dams were regrouped into 5 animals per box, the same ones prior to separation. At the end of pregnancy, in the afternoon of GD 21, all dams were taken to the Maternity of Physiology Laboratory (1 animal per box), where parturition occurred. During lactation, there was no manipulation of the offspring, except for box exchange (3 times a week), performed with a plastic shovel to avoid contact between the pups and the experimenter. At postnatal day 21 the pups were weaned and, in a separate room, sexed and identified by different pen marks in their tails. After that, male progeny was conducted to Male’s Vivarium of Physiology Laboratory.

### Offspring and experimental groups

In Male’s Vivarium (same conditions of temperature and luminosity of Female’s Vivarium), the male pups were grouped into 5 animals per box (polypropylene, 32 x 38 x 18 cm, lined with sawdust) according to the gestational week in which their dams were socially separated (whenever possible, siblings were not allocated in the same box. To avoid ‘litter effect’, at most 3 pups from the same dam were used in the same group). In this way, 4 large groups were formed: ***Control Group*** (pups from dams who were not separated at any time during gestation period), ***1***^***st***^ ***Gestational Week Group– 1***^***st***^ ***GW*** (pups from dams socially separated during 1^st^ gestational week), ***2***^***nd***^ ***Gestational Week Group– 2***^***nd***^ ***GW*** (pups from dams socially separated during 2^nd^ gestational week) and ***3***^***rd***^ ***Gestational Week Group– 3***^***rd***^ ***GW*** (pups from dams socially separated during 3^rd^ gestational week). The pups were still divided into 3 subgroups, according to the month of life in which they were submitted to behavioral assessment: ***1***^***st***^**, *3***^***rd***^ ***and 6***^***th***^ ***month*.** Once in Male’s Vivarium, the animals received water and chow *ad libitum* and were not handled until the suitable age for assessment, except for exchange of the boxes (3 times a week).

### Behavioral assessment

Being within the suitable month for the assessment [1^st^ month: age between 31 and 40 days old–n(control) = 9, n(1^st^ GW) = 10, n(2^nd^ GW) = 9, n(3^rd^ GW) = 9]; [3^rd^ month: age between 91 and 98 days old–n(control) = 10, n (1^st^ GW) = 10, n(2^nd^ GW) = 9, n(3^rd^ GW) = 10]; [6^th^ month: age between 181 and 186 days old–n(control) = 7, n(1^st^ GW) = 9, n(2^nd^ GW) = 8, n(3^rd^ GW) = 9], males were evaluated in ETM. As already mentioned, ETM was developed by Graeff and colleagues [33, 34] by removing one of the enclosed arms of EPM and seeks to generate in a same animal two defensive responses: inhibitory avoidance and escape, that have been related, respectively, to the generalized anxiety disorder and panic [33, 34, 36–39].

It was used an ETM made of wood, consisting of three arms of same dimensions (50 x 12 cm), elevated 50 cm from the floor [33]. One of the arms was surrounded by lateral walls (40 cm tall) and perpendicular to the two open arms. Open arms were delimited by a transparent acrylic protection of 1 cm height to prevent the fall of animals. After 3 consecutive days of handling (5 minutes/day), twenty-four hours before the behavioral assessment, the animals were kept individually, for thirty minutes, in one of the open arms of ETM (pre-exposure). For this, open arms were separated by a wooden wall, disposed on the line between the central area of the maze and the proximal portion of the arm open. After this procedure, animals were returned to their boxes until the next day.

The test in ETM was initiated by inhibitory avoidance measurement. Each animal was placed at the distal end of the enclosed arm facing the intersection of the arms. The time taken by the rat to leave this arm with the four paws towards the center of the apparatus was recorded (Baseline latency). The same measurement was repeated in two subsequent trials (Avoidance 1 and 2) at 30 seconds intervals, during which animals were placed in a polypropylene box with sawdust. Thirty seconds after the last avoidance, the rat was placed at the end of the same open arm used in the pre-exposure session and the time taken to leave this arm with the four paws was recorded in three consecutive trials (Escape 1 to 3), again with 30 seconds intertrial intervals. A cutoff time of 300 seconds was established for avoidance and escape latencies. After test, the number of fecal boli was counted. Thirty seconds after being tested in ETM, animals were individually placed in the center of an open-field for the evaluation of locomotor activity, where the total number of squares crossed by the animal was counted for 5 minutes. The open-field was made of wood, measuring 60 x 60 cm, with marked squares of 20 x 20 cm.

The tests were carried out in an exclusive room for behavioral assessment of Physiology Laboratory (temperature of 21°C ± 2°C), between 2 and 5 p.m. During the assessment, each rat was taken individually in a polypropylene box lined with sawdust to the experimental room. The apparatuses were illuminated by an incandescent light bulb attached to the roof, providing a luminous intensity of 50 lux, avoiding shadows in any one of the arms. The apparatuses were cleaned with a 20% ethanol solution after the end of test of each animal. When there were fecal and/or urinary excretion, ETM was also cleaned before the next trial of the same animal. The experimenter stayed outside the room, tests were video recorded and analyzed through the program Etholog 2.25 [45]. Immediately after testing in ETM and in open-field, each animal separately was sacrificed by decapitation (in another room for this purpose, in total control of the noise, luminosity, handling, using all procedures to alleviate suffering of the animals, without any contact/communication with others rats). Brain, thymus, spleen, adrenals and reproductive system were removed for further analysis (to be published).

### Statistical analysis

All data were analyzed using the software Statistica 6.0 (Statsoft). The analyses were carried out month by month (1^st^, 3^rd^ or 6^th^ month). For ETM, to assess if there was learning during the avoidances or changes between the escapes trials within the same group, it was used ANOVA of repeated measures. Separately, for the analysis of each trial of avoidance (Baseline, Avoidance 1 or Avoidance 2) or escape (Escape 1 to 3) it was used one-way ANOVA, where control animals were evaluated according to the month of life (comparison between 1^st^, 3^rd^ and 6^th^ month) and experimental groups (3 groups—animals whose dams were socially separated during 1^st^, 2^nd^ e 3^rd^ gestational week) were compared to control groups, also according to the month of life analyzed. The number of crossed squares in open-field, as well as the animals' body weight and total fecal boli eliminated in ETM were analyzed by one-way ANOVA. *Post-hoc* comparisons were made through Duncan’s test. In all analysis, a value of p<0.05 was considered significant.

## Results

### Control animals—behavioral and physiological results

**Fig 1:**
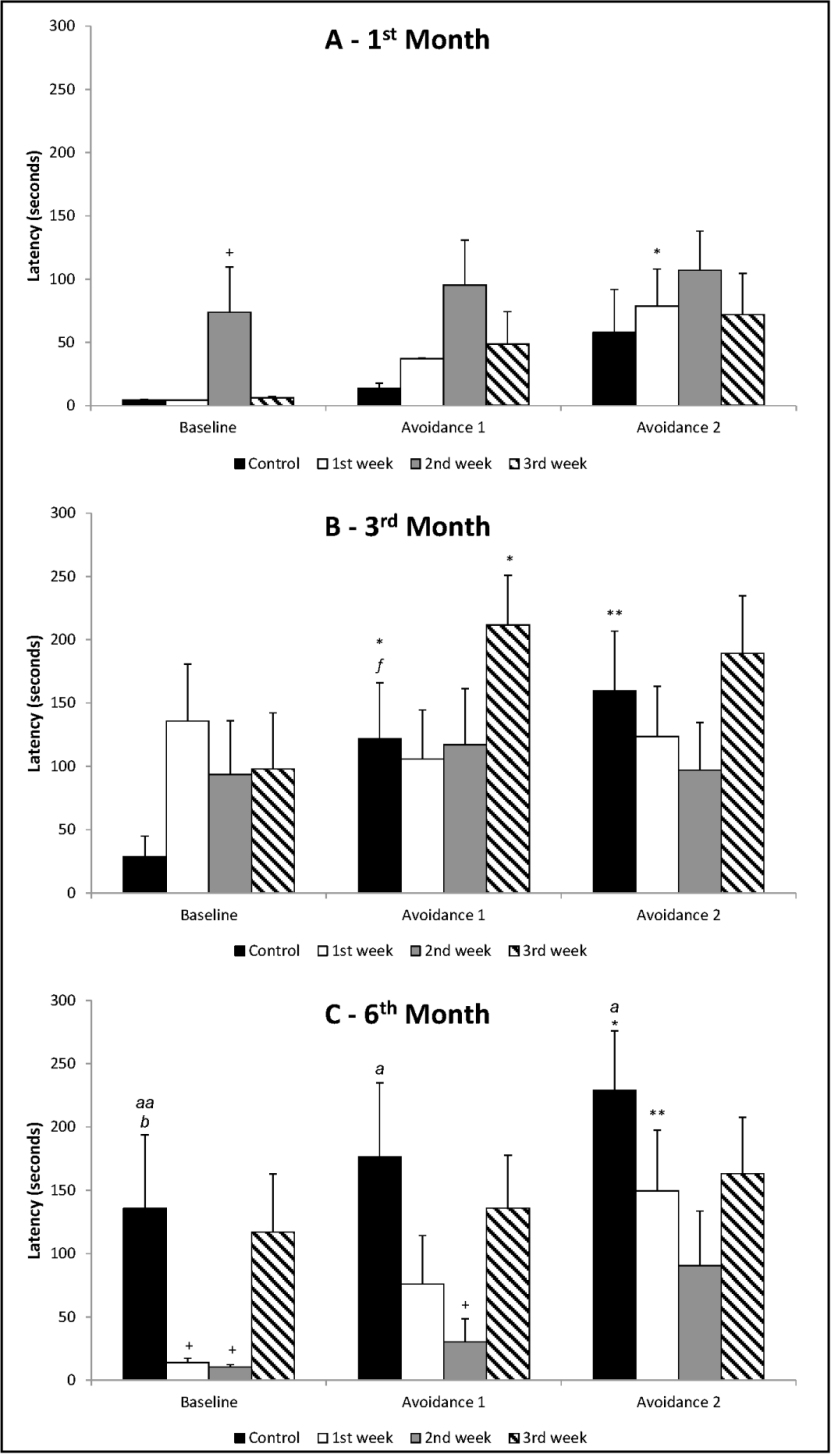
**(A) 1**^**st**^ **month of life; (B) 3**^**rd**^ **month of life; (C) 6**^**th**^ **month of life.** Mean + SEM of latency in seconds for avoidances in ETM. *Learning*: ANOVA of repeated measures followed by Duncan’s test, where: *p<0.05 and **p<0.01 in relation to Baseline of the same group within the evaluated month. *Differences between groups within the same month*: one-way ANOVA followed by Duncan’s test, where: ^+^p<0.05 in relation to the same trial of control group. *Comparison among control groups (AxBxC)*: one-way ANOVA followed by Duncan’s test, where: ^*a*^p<0.05 and ^aa^p<0.01 in relation to the same trial of 1^st^ month; ^*b*^p<0.05 in relation to the same trial of 3^rd^ month. *f* indicates a trend result in relation to the same trial of 1^st^ month.

In the analysis between avoidances in control rats (Baseline, Avoidance 1 and Avoidance 2), ANOVA of repeated measures showed that there were differences between the months [F_(2,23)_ = 6.46; p = 0.006] and effect of trials [F_(2,46)_ = 7.16; p = 0.002], but without interaction between months and trials [F_(4,46)_ = 0.70; p = 0.599] (Fig 1A, Fig 1B and Fig 1C). The *post-hoc* test (Duncan’s test) pointed out that animals in the 3^rd^ month of life presented an increase of Avoidance 1 and 2 in relation to Baseline (p<0.05 and p<0.01, respectively). The same was observed in animals of 6 months (increased Avoidance 2 in relation to Baseline; p<0.05), i.e., there was an increase of avoidances throughout the trials, featuring a defense behavioral repertoire, as expected for control groups. The differences were not significant in the analysis of animals in the 1^st^ month of life. Analyzing each trial separately, one-way ANOVA showed that there was difference between the months for Baseline [F_(2,23)_ = 5.20; p = 0.014]. Duncan’s test pointed out that animals in the 6^th^ month of life showed increase of the avoidance in relation to animals at 1^st^ (p<0.01) and at 3^rd^ month (p<0.05). Regarding Avoidance 1 and 2 there were differences between the months, respectively [F_(2,23)_ = 4.10; p = 0.030] and [F_(2,23)_ = 3.65; p = 0.042]. The analysis by Duncan’s test showed that animals in 3^rd^ month of life showed a trend increase in the Avoidance 1 (p = 0.070) compared to animals in 1^st^ month. Even so, the animals in 6^th^ month showed an increase in the Avoidance 1 (p<0.05), as well as Avoidance 2 (p<0.05), when compared to animals of the 1^st^ month. These data indicate that the manifestation of behaviors associated with generalized anxiety disorder increased in older control animals, whose dams did not experience the impact of social separation during pregnancy.

**Fig 2:**
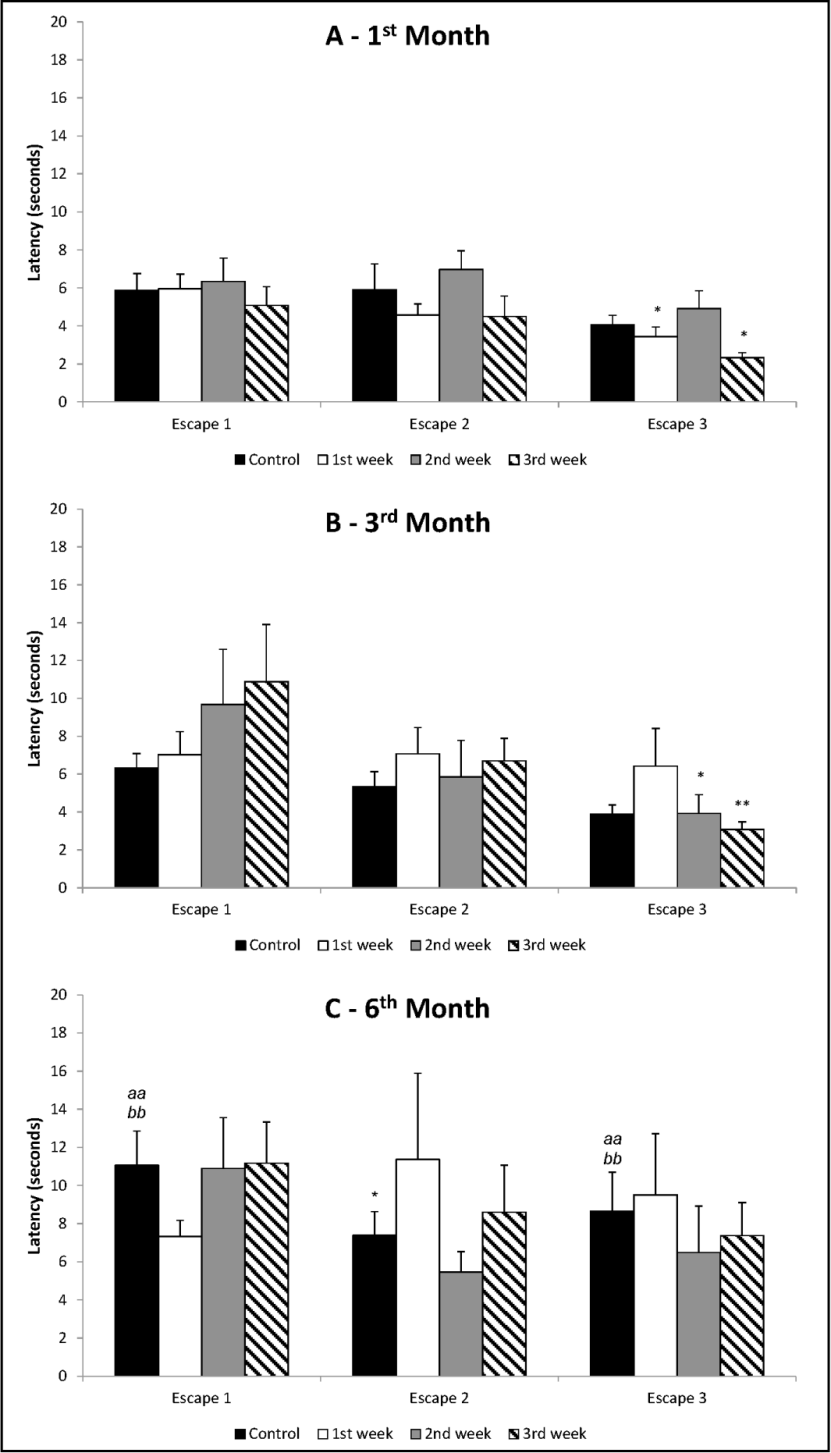
**(A) 1**^**st**^ **month of life; (B) 3**^**rd**^ **month of life; (C) 6**^**th**^ **month of life.** Mean + SEM of latency in seconds for escapes in ETM. *Changes among escapes*: ANOVA of repeated measures followed by Duncan’s test, where: *p<0.05 and **p<0.01 in relation to Escape 1 of the same group within the evaluated month. *Differences between groups within the same month*: one-way ANOVA followed by Duncan’s test (there were no differences). *Comparison among control groups (AxBxC)*: one-way ANOVA followed by Duncan’s test, where: ^*aa*^p<0.01 in relation to the same trial of 1^st^ month; ^*bb*^p<0.01 n relation to the same trial of 3^rd^ month.

Regarding the escapes presented by control animals (Fig 2A, Fig 2B and Fig 2C), the ANOVA of repeated measures showed that there were differences between the months [F_(2,23)_ = 8.01; p = 0.002] and effect of trials [F_(2,46)_ = 3.95; p = 0.026], without interaction between months and trials [F_(4,46)_ = 1.04; p = 0.399]. There were no differences between the 3 trials of animals in 1^st^ and 3^rd^ month of life. However, in 6^th^ month there was a reduction of latency for Escape 2 compared to Escape 1 (Duncan’s test: p<0.05). Analyzing each trial separately, one-way ANOVA showed that there was difference between the months for Escape 1 [F_(2,23)_ = 6.06; p = 0.008]. There was an increase of latency for leaving open arm for animals in the 6^th^ month, in relation to animals in the 1^st^ (Duncan’s test: p<0.01) and 3^rd^ month of life (Duncan’s test: p<0.01). In relation to Escape 2, there were no differences between the months [F_(2,23)_ = 0.83; p = 0.450]. In Escape 3, there were differences between the months [F_(2,23)_ = 6.13; p = 0.007]: there was an increased latency to leave the open arm for animals assed in 6^th^ month, when compared to animals in 1^st^ and 3^rd^ month of life (Duncan’s test: p<0.05). These data indicate that the manifestation of panic-like behaviors decreased in older control rats.

**Fig 3:**
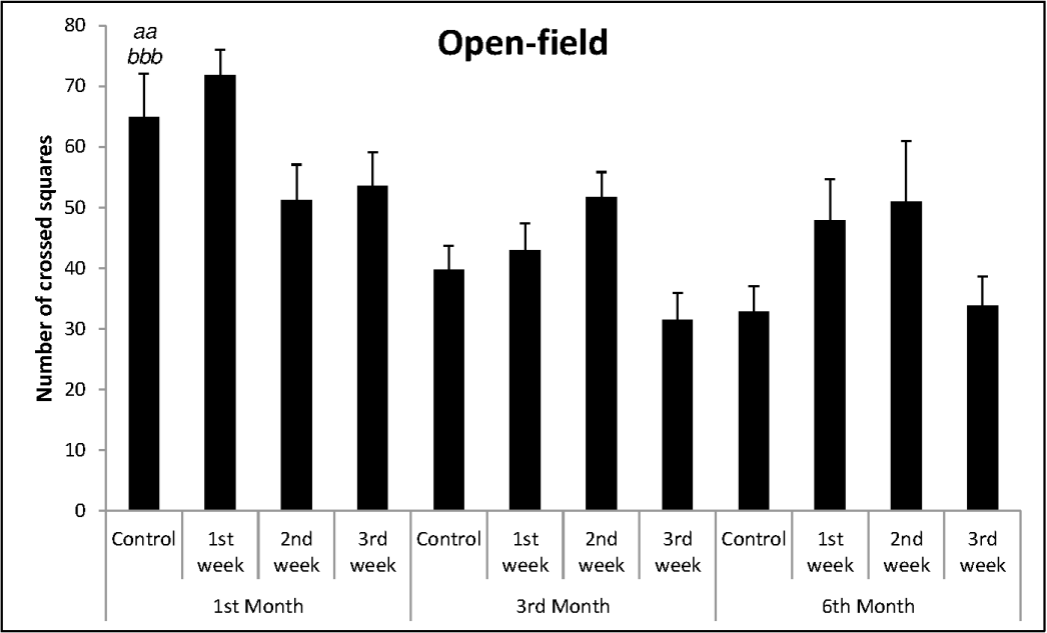
Mean + SEM of number of crossed squares in open-field. One-way ANOVA followed by Duncan’s test, where: ^*aa*^p<0.01; ^*bbb*^p<0.001, when compared to control group of 3^rd^ and 6^th^ month, respectively.

Analyzing the number of crossed squares on open-field by control animals (Fig 3), one-way ANOVA showed difference between the months [F_(2,23)_ = 9.47; p = 0.001]. It was found an increase in the number of crossed squares in 1^st^ month of life in relation to 3 and 6 months old animals (Duncan’s test: p<0.01 and p<0.001, respectively), indicating increased locomotor activity in early development.

**Fig 4:**
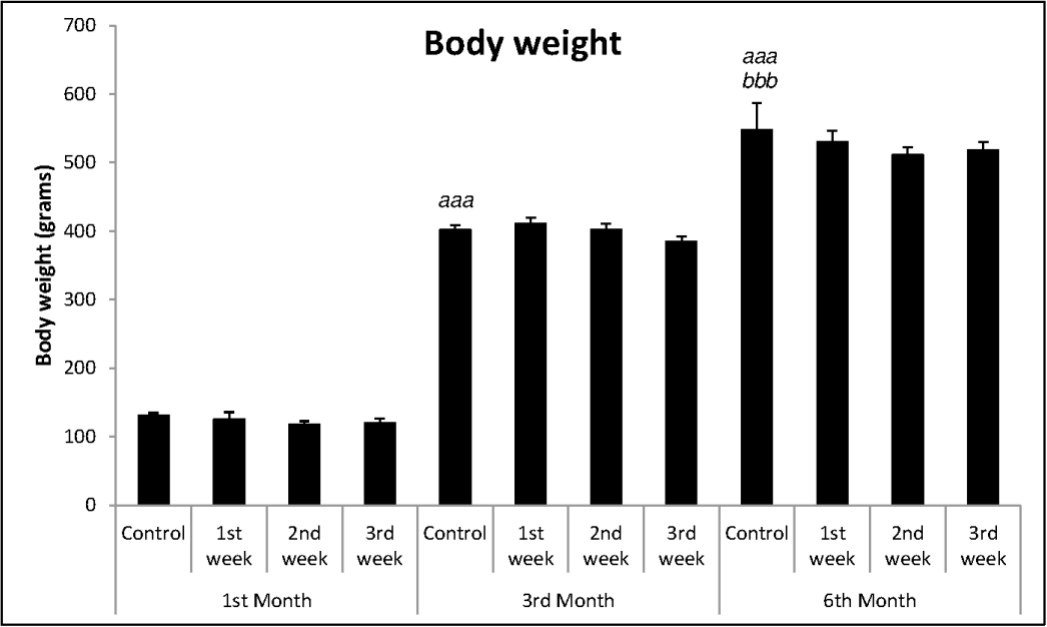
Mean + SEM of body weight in grams. One-way ANOVA followed by Duncan’s test, where: ^*aaa*^p<0.001 in relation to control group of 1^st^ month and ^*bbb*^p<0.001 in relation to control group of 3^rd^ month.

In the analysis of body weight (Fig 4), one-way ANOVA showed differences between the months [F_(2,23)_ = 118.64; p<0.001]. Animals of 6 months were heavier than the animals of 1 and 3 months (p<0.001 for both) and animals of 3 months, heavier than 1 month’s ones (p<0.001).

**Fig 5:**
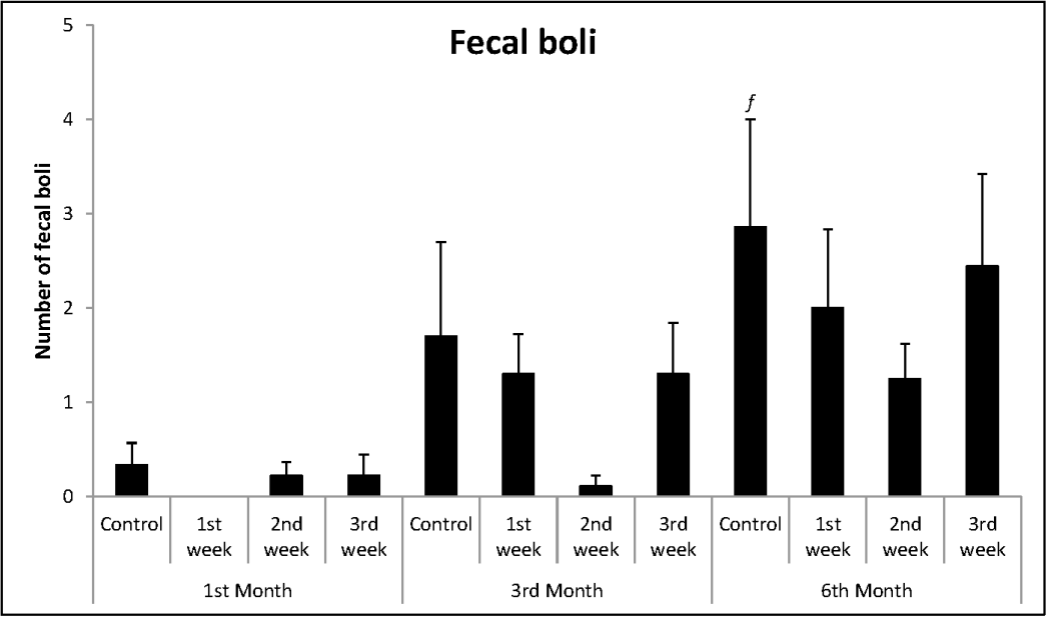
Mean + SEM of number of fecal boli eliminated on ETM.

For the number of fecal boli excreted in ETM (Fig 5), one-way ANOVA showed that there were no differences between the months [F_(2,23)_ = 1.97; p = 0.162].

### Effect of dams’ exposure to social separation in different gestational weeks on the behavioral and physiological variables of the offspring in 3 distinct stages of development (1^st^, 3^rd^, and 6^th^ month of life)

#### 1^st^ month of life

Analyzing the avoidances presented by animals in the 1^st^ month of life (Fig 1A), ANOVA of repeated measures showed that there were no differences between groups [F_(3,33)_ = 2.02; p = 0.130], but there was effect of trials [F_(2,66)_ = 7.18; p = 0.002] without interaction between groups and trials [F_(6,66)_ = 0.28; p = 0.943]. Duncan’s test revealed an increase of time in Avoidance 2 compared to Baseline for animals whose dams were socially separated during 1^st^ gestational week (p<0.05). That is, the offspring whose mothers were separated in the 2^nd^ and 3^rd^ week of gestation did not present acquisition of avoidances. In the analysis of each trial separately, one-way ANOVA showed that, for Baseline, there were differences between groups [F_(3,33)_ = 3.83; p = 0.019], but the same was not observed for Avoidance 1 [F_(3,33)_ = 1.63; p = 0.202] and Avoidance 2 [F_(3,33)_ = 0.38; p = 0.770]. It was found increased latency on Baseline presented by animals whose dams were socially separated during 2^nd^ gestational week, compared to the control group (Duncan’s test: p<0.05). This result indicates that social separation during 2^nd^ gestational week caused a raise of anxiety-like behaviors in animals in 1^st^ month of life, predisposing them to the manifestation of generalized anxiety disorder.

Regarding the escapes (Fig 2A), ANOVA of repeated measures showed that there were no differences between groups [F_(3,33)_ = 1.85; p = 0.158], but there was effect of trials [F_(2,66)_ = 10.02; p<0.001], without interaction between groups and trials [F_(6,66)_ = 0.43; p = 0.857]. Animals whose dams were socially separated during 1^st^ or 3^rd^ gestational week left open arm faster along trials, i.e., there was a decrease of time for the escapes, with reduction of time for Escape 3 to Escape 1 (Duncan’s test: p<0.05). In the analysis of each trial singly, one-way ANOVA showed no differences between the groups for Escape 1 [F_(3,33)_ = 0.29; p = 0.831] and Escape 2 [F_(3,33)_ = 1.35; p = 0.276] and although there were differences when we examined Escape 3 [F_(3,33)_ = 3.18; p = 0.037], Duncan's test did not reveal alterations between animals from control dams and animals from dams socially separated in one of the gestational weeks.

One-way ANOVA showed differences in the number of crossed squares between groups [F_(3,33)_ = 2.92; p = 0.049], but no relevant differences were found between animals from control dams and those ones from dams socially separated in one of the gestational weeks (Fig 3).

There were no differences between groups neither in the analysis of the body weight (Fig 4) [F_(3,33)_ = 0.67; p = 0.576], nor in the evaluation of fecal boli [F_(3,33)_ = 0.68; p = 0.570], as shown in Fig 5.

#### 3^rd^ month of life

Concerning the avoidances (Fig 1B), ANOVA of repeated measures showed no differences between groups [F_(3,35)_ = 0.85; p = 0.474], but there was effect of trials [F_(2,70)_ = 3.80; p = 0.027], without interaction between groups and trials [F_(6,70)_ = 1.78; p = 0.115]. Dams socially separated during 1^st^ or 2^nd^ gestational week generated animals that in the 3^rd^ month of life did not learn to avoid aversive situation. Learning of avoidances was evidenced only in animals whose dams were socially separated during 3^rd^ gestational week (increase of Avoidance 1 in relation to Baseline; Duncan’s test: p<0.05). In the analysis of each trial, one-way ANOVA showed that there were no differences between groups for Baseline [F_(3,35)_ = 1.35; p = 0.273], Avoidance 1 [F_(3,35)_ = 1.42; p = 0.254] and Avoidance 2 [F_(3,35)_ = 0.87; p = 0.465].

Regarding the escapes (Fig 2B), ANOVA of repeated measures showed that there were no differences between groups [F_(3,35)_ = 0.52; p = 0.669], but there was effect of trials [F_(2,70)_ = 8.28; p = 0.001], without interaction between groups and trials [F_(6,70)_ = 1.34; p = 0.250]. Duncan’s test showed that animals whose dams were socially separated during 2^nd^ or 3^rd^ gestational week presented a reduction of time along trials (Escape 3 compared to Escape 1, p<0.05 and p<0.01, respectively). In the analysis of each escape, one-way ANOVA showed no differences between groups in any of the trials: Escape 1 [F_(3,35)_ = 1.01; p = 0.402], Escape 2 [F_(3,35)_ = 0.35; p = 0.792] and Escape 3 [F_(3,35)_ = 1.59; p = 0.210].

One-way ANOVA showed that there were differences in relation to motor activity in the open-field [F_(3,35)_ = 3.80; p = 0.018]. However, Duncan’s test did not show differences between the groups whose dams were socially separated and control group, as shown in Fig 3.

In the analysis of body weight (Fig 4), one-way ANOVA showed that there were no differences between groups [F_(3,35)_ = 1.82; p = 0.162]. Similarly, no differences were found in the analysis of fecal boli elimination in ETM [F_(3,35)_ = 1.15; p = 0.341] (Fig 5).

#### 6^th^ month of life

When we analyzed the avoidances in 6^th^ month of life (Fig 1C), ANOVA of repeated measures showed that there were differences between groups [F_(3,29)_ = 3.27; p = 0.035] and effect of trials [F_(2,58)_ = 8.70; p<0.001], but without interaction between groups and trials [F(6,58) = 0.43; p = 0.856]. Duncan's test revealed learning occurred only in animals whose dams were socially separated during 1^st^ gestational week, with increased time of Avoidance 2 in relation to Baseline (p<0.01). There was no learning in animals whose dams were socially separated during 2^nd^ or 3^rd^ gestational week. Analyzing each trial, one-way ANOVA showed differences for Baseline [F_(3,29)_ = 3.49; p = 0.028]: it was found a decrease in latency of animals whose dams were socially separated during 1^st^ or 2^nd^ gestational week (Duncan’s test: p<0.05) in relation to control group. Although one-way ANOVA showed no differences for Avoidance 1 [F_(3,29)_ = 2.38; p = 0.090] and Avoidance 2 [F(3,29) = 1.39; p = 0.267]. Duncan’s test revealed decrease of time for Avoidance 1 in the group of animals whose dams were socially separated during 2^nd^ gestational week (p = 0.025) compared to the control group. The reduction of time of Baseline and/or Avoidance 1 shows that animals whose dams were socially separated during 1^st^ or 2^nd^ gestational week presented a decreased of behaviors associated with anxiety generalized disorder.

Regarding the escapes (Fig 2C), ANOVA of repeated measures showed no differences between the groups [F_(3,29)_ = 0.17; p = 0.918], neither effect of trials [F_(2,58)_ = 1.50; p = 0.232], nor interaction between groups and trials [F_(6,58)_ = 1.40; p = 0.231]. Similarly, in the analysis of each trial, one-way ANOVA showed no differences between groups for Escape 1 [F_(3,29)_ = 0.97; p = 0.421], neither Escape 2 [F_(3,29)_ = 0.74; p = 0.536], nor Escape 3 [F_(3,29)_ = 0.30; p = 0.825].

In relation to crossed squares in the open-field (Fig 3), one-way ANOVA showed that there were no differences between groups [F_(3,29)_ = 1.84; p = 0.162]. The same was observed in relation to body weight [F_(3,29)_ = 0.53; p = 0.663] and to fecal boli elimination [F_(3,29)_ = 0.59; p = 0.629], according to Figs 4 and 5, respectively.

## Discussion

Observing only control groups it was possible to verify changes in manifestation of the defensive behaviors related to anxiety along the development of rats whose dams were kept in groups during the gestational period. Knowing that puberty in male rats starts about 46 days old [46], animals evaluated within the 1^st^ month of life were considered pre-pubescent, while animals evaluated within the 3^rd^ and 6^th^ month were considered young adults and mature adults, respectively. The expected behavior of an animal assessed in ETM is that there is learning of avoidances, i.e., that the latency to leave the enclosed arm increases along the 3 trials (Baseline, Avoidance 1 and Avoidance 2), and that there are no changes in escapes, i.e., that the latency to leave the open arm remains constant along the 3 trials (Escape 1 to 3) [33, 34, 36–39]. Learning of avoidances was observed in control animals of 3 and 6 months, but not in those ones in the 1^st^ month of life, showing that, under normal conditions, a certain degree of maturity is required for the occurrence of learning.

The results showed that in 6^th^ month of life there was an increase of latencies both to avoidances and escapes of rats whose dams were not subjected to any kind of stressor during gestation, i.e., with the advancing age there were manifestation of behaviors related to generalized anxiety disorder and decreased panic-related behaviors. When it comes to anxiety, these results are consistent with previous investigations that found a raise of this disorder throughout male rats’ life, characterized by lower exploration of open arms in EPM [47–49].

Animals in 6^th^ month of life also showed reduced locomotor activity and increased body weight. One can ask if the reduction in locomotor activity, also potentiated by the increase of body weight, would have caused the rats to remain longer in enclosed and open arms, raising the latencies for avoidance and escape, respectively. Meanwhile, in this analysis, it is appropriate to emphasize that the differences found were in relation to animals in 1^st^ month, presenting a striking increase in motor activity, and not in relation to adult animals in the 3^rd^ month. Andrade and colleagues [47] have also found greater motor activity in rats of 1 month and its reduction in the 6^th^ month, however using the EPM (number of closed arms entries), corroborating the present study. As for the body weight, 6 months animals were heavier compared to those ones of 1 and 3 months. Nevertheless, rats with 6 months, generated by dams that were socially separated during 1^st^ or 2^nd^ gestational week, showed the same body weight of control ones with the same age, and the same range of crossed squares in open-field, but their responses in ETM were quite different, showing impairment in the avoidances, i.e., they left the enclosed arms faster. In other words, in this case there was no influence of any kind of locomotor activity and weight over the behavioral test performance. Therefore, it is possible to affirm that the behavior in ETM, for control animals with 6 months of age, was not affected by locomotor activity and by weight of the animals.

As for animals whose dams were socially separated in one of the three gestational weeks, there was variation in the learning of avoidances and in changes of escapes, when we compared the gestational week in which the separation occurred with different stages of offspring development. Regarding the animals in 1^st^ month of life, learning of avoidances only occurred for those ones whose dams were separated during 1^st^ gestational week. Dams socially separated during 1^st^ or 2^nd^ gestational week generated a progeny who did not learn to avoid aversive situations in 3^rd^ month of life. At this stage, learning was only evidenced in animals whose dams were socially separated during 3^rd^ gestational week. On the other hand, in 6^th^ month, rats whose dams were socially separated during 1^st^ gestational week showed learning, but the same was not observed for the ones whose dams were separated during 2^nd^ or 3^rd^ week. In the latter case, Baseline of animals was already high. These data indicate that both variables, week of prenatal stress exposure and the developmental stage of the progeny, affect the aversive conditioning. However, social separation during 2^nd^ gestational week was more powerful to the impairment of learning, as the offspring of dams subjected to this stressor during that week does not presented learning of avoidances in any of the evaluated periods.

About the effect of prenatal stressors on the manifestation of progeny’s anxiety, the results showed that social separation during 2^nd^ week of gestation caused increase in latency of avoidance for 1 month old animals, predisposing them to the manifestation of generalized anxiety disorder. Still, the same type of aversive experience, but during 1^st^ or 3^rd^ gestational week, caused changes among the escapes in the offspring with that same age, showing reduced latency along the trials of escape, an effect interpreted in this study as panicogenic. These results demonstrate that the gestational period in which occurred the exposure to aversive experience differentially affects the kind of anxiety manifested in the offspring in 1^st^ month of life.

On the other hand, the stress of social separation experienced by dams during 1^st^ or 2^nd^ week of pregnancy affected the response of the progeny in its 6^th^ month of life, but in a reverse way than expected: there was an impairment of avoidances, *i*.*e*., these animals left the enclosed arms faster than control ones, an anxiolytic-like effect. Some researchers have discussed this aspect, justifying that when there is a demand for the prenatally stressed offspring, it responds to aversive stimuli in a more adaptive way [50]. This could be a line of reasoning. However, this aspect has only been found in an advanced period of adulthood, when may also exist morphofunctional changes in the structures and pathways that regulate anxiety.

In rats, most of the studies of prenatal stress neurotoxicity are performed using the stressor during 3^rd^ gestational week. However, a work of Xu and colleagues [51] has shown that prenatal stress on 2^nd^ and 3^rd^ gestational weeks was more forceful than when applied only in the 3^rd^ gestational week, causing greater neuronal degeneration, structural alterations in myelin (critical for nerve conduction) and reduced efficiency in synaptic transmission in the hippocampus. In relation to the present study, perhaps the changes observed in animals whose dams were separated during 1^st^ or 2^nd^ gestational week are arising from the bigger impairment in the developing of fetus’ brain in early stages of pregnancy.

The established paradigm for the research of the effects of prenatal stress by applying the stressor from gestational day 15 to 21 is based on the fact that during this period the hippocampus of the fetus is developing and its HPA axis starts to secrete its own ACTH and corticosterone [51]. Besides, glucocorticoid receptor mRNA can only be detected in the rat brain from gestational day 13 and changes in maternal steroid levels occurring after that day could influence neuronal activity and HPA axis function in the offspring, by interacting with glucocorticoid receptors [1]. However, the development of central nervous system includes a number of processes started in sequence and which are dependent of each other in many ways. So, if dams are exposed to stress before gestational day 15, although the hippocampus of fetus is not formed and HPA axis has not started its activity, such stress may have an effect on a pre-existing neuroendocrine or remodeling variation in an early developmental stage and so might affect subsequent stages of development. Therefore, when the exposure occurs in initial stages of pregnancy, a greater prejudice in the brain development of the fetus would be expected [51]. In the present study, we found effects of prenatal stress when it was applied both in early and later stages of pregnancy.

## Conclusions

The psychological changes produced by maternal stress, social separation in this approach, clearly depend on the stage of development of the offspring, so that gestational period of exposure to aversive experience differentially affects the kind of anxiety manifested throughout postnatal life. In the present study it was observed that prenatal stress effects occurred at every gestational week of rats, especially during the 2^nd^ one. The results reinforce that stress in critical periods of embryonic or fetal development can alter brain programming, increasing the susceptibility to psychopathologies in offspring, like different types of anxiety.

## Supporting information

S1 File Dataset.xlsx. (XLSX)
